# MapReduce based big data framework using associative Kruskal poly Kernel classifier for diabetic disease prediction

**DOI:** 10.1016/j.mex.2025.103210

**Published:** 2025-02-05

**Authors:** R. Ramani, S. Edwin Raja, D. Dhinakaran, S. Jagan, G. Prabaharan

**Affiliations:** a Department of Artificial Intelligence and Data Science, P.S.R Engineering College, Sivakasi, India; b Department of Computer Science and Engineering, Vel Tech Rangarajan Dr. Sagunthala R&D Institute of Science and Technology, Chennai, India

**Keywords:** Machine learning, Associative Kruskal Wallis, MapReduce, Bigdata, Poly Kernel, Associative Kruskal Wallis and MapReduce Poly Kernel

## Abstract

Recent trendy applications of Artificial Intelligence are Machine Learning (ML) algorithms, which have been extensively utilized for processes like pattern recognition, object classification, effective prediction of disease etc. However, ML techniques are reasonable solutions to computation methods and modeling, especially when the data size is enormous. These facts are established due to the reason that big data field has received considerable attention from both the industrial experts and academicians. The computation process must be accelerated to achieve early disease prediction in order to accomplish the prospects of ML for big data applications. In this paper, a method named “Associative Kruskal Wallis and MapReduce Poly Kernel (AKW-MRPK)" is presented for early disease prediction. Initially, significant attributes are selected by applying Associative Kruskal Wallis Feature Selection model. This study parallelizes polynomial kernel vector using MapReduce based on the significant qualities gained, which will become a significant computing model to facilitate the early prognosis of disease. The proposed AKW-MRPK framework achieves up to 92 % accuracy, reduces computational time to as low as 0.875 ms for 25 patients, and demonstrates superior speedup efficiency with a value of 1.9 ms using two computational nodes, consistently outperforming supervised machine learning algorithms and Hadoop-based clusters across these critical metrics.

• The AKW-MRPK method selects attributes and accelerates computations for predictions.
• Parallelizing polynomial kernels improves accuracy and speed in healthcare data analysis.

## Graphical abstract

**Figure:**
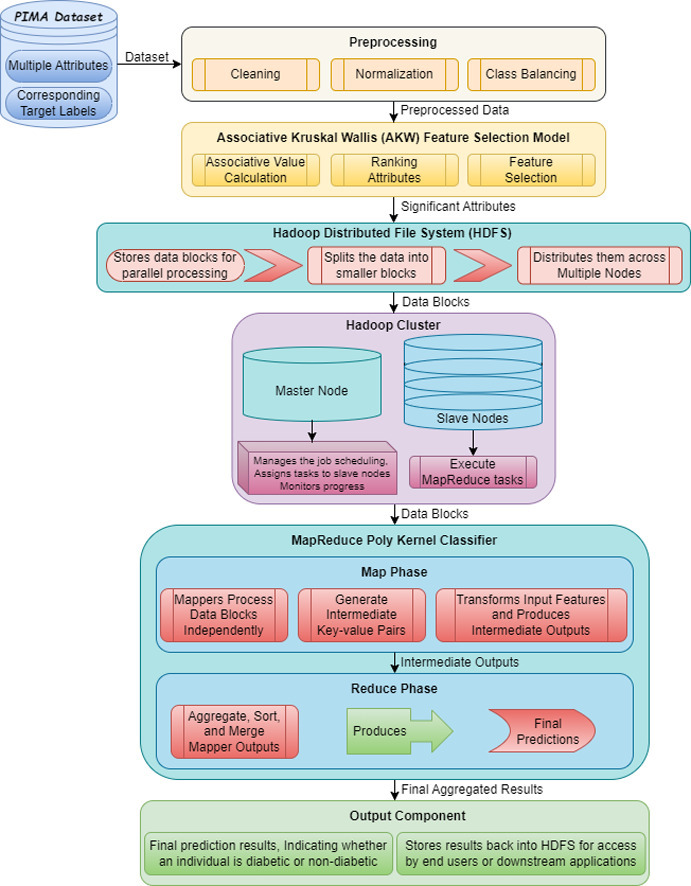

Specifications table

**Table:** 

Subject area:	Engineering
More specific subject area:	MapReduce Based Big Data Framework for Diabetic Disease Prediction
Name of your method:	Associative Kruskal Wallis and MapReduce Poly Kernel
Name and reference of original method:	Machine Learning, Associative Kruskal Wallis, MapReduce, Poly Kernel.
Resource availability:	N*.A.*

## Introduction

One of the most common metabolic diseases faced in this era is diabetes. The most common fact is that the occurrence of type 2 diabetes is usually found in either the middle age or in case of old age. But, in recent days, even children are the victims of diabetes [1]. Several factors are said to be the reasons for developing diabetes irrespective of factors like genetic susceptibility, excessive body weight, high blood pressure, change in the food consumption patterns, lifestyle etc. If diabetes is not said to be diagnosed in the early stages, it will cause several complications like increased blood sugar, resulting in diabetic retinopathy, glaucoma, foot ulcer etc. [2]. Hence, early diabetes detection is highly necessitated to improve patient quality of lifestyle and in turn improving life expectancy. Harleen Kaur and Vinita Kumari [3] proposed supervised machine learning algorithms for classification of patients into diabetic and non-diabetic categories. First, irrelevant and inconsistent data were handled by applying Boruta Wrapper algorithm. Using this algorithm, unbiased feature selection was ensured. Followed by which five different supervised machine learning algorithms were applied to the selected attributes to perform binary classification. Thus, with the limited number of parameters, both accuracy and precision are achieved. However, the time involved in the features selection was not focused. In the research contribution of this paper, significant features that form the basis for classification are selected using dual factors, namely ‘’Associative value and Kruskal Ranking’’ to reduce the time involved.

Yuvaraj and Sri Preethaa [4] proposed machine learning algorithms in Hadoop-based clusters for the prediction of diabetic that provided both the processing as well as the storing of large datasets. To start with, information gain was used as the feature selection technique. Subsequently, machine learning algorithm R was integrated into Hadoop, resulting in highly accurate diabetes predictive mechanism [5]. Though accuracy was said to be attained via R machine learning, however, the rate at which the prediction was not analyzed for the scalable training dataset. In our proposed work, a MapReduce Poly Kernel Vector Classifier model was used to address this issue. Even with the increasing volume of the training dataset (i.e., big data), the speedup is said to improve along with the precision and accuracy. The motivation of this work includes healthcare associations produces huge amount of data from different sources namely clinical data, diagnosis data and doctor instruction and they are unorganized. Healthcare industry experiences lot of demands. Healthcare sector produces data in the form of text, image, audio, video and Electronic Health Record (EHR) which expose the significance to implement data analytics and shape up big data in healthcare. Diabetes mellitus is chronic disease caused, due to high sugar level in the circulatory system [6]. Analysis of big data produces predicted results for better recognition of trends in enhancing the healthcare and life expectancy in order to provide effective treatment at the early stages. Prediction of data analytics for future events is performed by means of machine learning approaches. Decision support System applies a number of machine learning techniques to predict the diseases easier by exploiting medical data sets.

### Diabetes and healthcare challenges

The healthcare community now faces diabetes mellitus Type 2 as one of the most widespread chronic health problems occurring throughout multiple population groups worldwide. Doctors traditionally detected diabetes mainly in senior people but currently this medical condition exists increasingly among young people from children up to adolescents. The worldwide number of individuals diagnosed with diabetes has dramatically increased throughout the last few years because obesity combined with bad nutrition habits and insufficient physical activity and natural hereditary factors. The World Health Organization (WHO) reports that diabetes kills millions worldwide each year while causing major obstacles for public health infrastructures around the world. The economic strain on healthcare programs intensifies because of diabetes because treatment costs pile up while overseeing both hospital stays and extended care. Patients with uncontrolled diabetes develop serious life-threatening complications which can include diabetic retinopathy alongside cardiovascular diseases kidney failure nerve damage and the possibility of needing limb amputations. Successful diabetes management with early detection has proven essential for stopping or postponing these medical complications. No obvious symptoms in the early stages of diabetes limit accurate diagnosis so researchers need data-driven systems for early prediction. Machine learning (ML) proves its value in medical detection systems thanks to its analytical processing abilities for big datasets which discover unseen patterns that enable early diagnosis expertise.

### Role of data analytics and machine learning in healthcare

The healthcare industry produces large volumes of data which includes electronic health records (EHR) as well as medical images and laboratory reports and many other types of data. The introduction of digital health technologies including wearables and mobile health applications has driven an unprecedented growth of healthcare data opening new predictive analysis possibilities in medical care. The combination of machine learning (ML) along with big data systems such as Hadoop shows exceptional value in processing large data volumes. Healthcare professionals use advanced algorithms to discover disease risk patients while enhancing treatment strategies and delivering better care to their patients. Numerous healthcare applications which use machine learning algorithms examine massive data collections to effectively diagnose and forecast different medical conditions like diabetes have displayed remarkable diagnostic capabilities. Through extensive training with big patient information datasets ML models reveal previously unknown patterns that help researchers identify important relationships. Supervised learning algorithms and their counterparts such as decision trees and support vector machines enable clinical practitioners to sort patients through multiple variables including blood sugar levels, age and body mass index thereby forecasting diabetes risk. Accurate medical diagnoses become challenging when class imbalance appears between different patient categories such as diabetic versus non-diabetic patterns. An unequal distribution between classes creates prediction challenges since the model will overreact to dominant classes while underperforming with the minority class. Through feature selection algorithms researchers achieve accurate model predictions by selecting the most important components that each contribute to precise predictions without bias. This leads to efficient decision support.

### Addressing big data challenges in healthcare

Healthcare datasets continue to expand in complexity alongside their growing size which makes data processing and analysis more difficult to manage. Standard machine learning systems fail to handle high-scale big data operation requirements effectively. By using Hadoop's MapReduce tools and other elements within its ecosystem healthcare providers can overcome data processing limitations through parallel processing of voluminous datasets across multiple network nodes. The processing speed increases significantly when MapReduce splits workloads into portions which run concurrently across nodes throughout the cluster. Healthcare organizations require quick processing of large datasets especially when they need to make decisions about early disease diagnosis or predictive modeling. Healthcare data volumes continue to expand rapidly which makes real-time data analysis essential for the industry. Machine learning algorithms working with Hadoop big data frameworks enable the upscaling of predictive models while improving their accuracy along the way. We apply advanced machine learning techniques with big data technologies to develop a diabetes prediction system. This work presents an innovative method which combines both feature selection techniques and parallel Hadoop processing together with a polynomial kernel support vector machine (SVM) model to achieve accurate predictions. With the Associative Kruskal Wallis (AKW) algorithm serving as our feature selection tool we can boost model accuracy while minimizing needed computational time across extensive datasets. Our methodology employs a MapReduce framework to handle big data effectively which addresses the difficulties of analysis at major scale that exist within medical data processing. Our work addresses the growing challenge of massive healthcare data combined with an increasing requirement for fast and precise predictive models. Our methodology seeks to enrich healthcare data analytics research by producing a versatile framework which healthcare providers can adapt to extend beyond diabetes prediction. Healthcare professionals obtain better patient outcomes through machine learning because it enables them to make informed decisions that enhance clinic practice alongside boosting diagnostic accuracy.

**Contributions of this Work:**

1. **Innovative Feature Selection Model:** Through the Associative Kruskal Wallis approach the research addressed class imbalance problems and achieved better computational speed in feature selection.
2. **Advanced Classification Framework:** The research created the MapReduce Poly Kernel Vector model that uses Hadoop clusters to analyze large-scale datasets by efficiently categorizing diabetic and non-diabetic features.
3. **Scalable Big Data Solution:** The framework combines MapReduce programming with distributed computing to manage big medical datasets securely while ensuring smooth performance and scale-up capabilities.
4. **Comprehensive Evaluation and Validation:** Essential testing confirmed how the proposed system performs better with accuracy, speed and scalability characteristics over classic and Hadoop-based machine learning approaches.
5. **Broader Applicability and Practical Utility:** The framework proved its universal application across multiple domains in medical diagnosis by showing abilities for use beyond diabetes diagnoses into cardiovascular diagnosis and oncology diagnosis fields.

The entire work has been presented in the following sections. The work in the field of diabetes prediction that uses MapReduce and Big Data is described in Section 2. The proposed Associative Kruskal Wallis and MapReduce Poly Kernel (AKW-MRPK) for early disease prediction is discussed in Section 3. The experimental setup and results are discussed in Section 4. In this part, further discussions and analysis is also provided. A summary of the work is given in section 5.

## Related work

Healthcare providers struggle to manage diabetes because the numerous biological and lifestyle factors that cause diabetes create a complex issue for diagnosis and treatment planning. Effective data processing becomes complicated because healthcare data contains large amounts and varied formats including structured forms such as lab results and unstructured documents like medical notes and images [7]. The varying nature of data leads to substantial challenges with incomplete datasets and additional unwanted data which creates barriers for precise evaluation. The major challenge in diabetes prediction involves skewed distribution of data because non-diabetic cases typically outnumber diabetic cases in available datasets. The distributional inequality between majority and minority classes distorts predictive models resulting in reduced ability to detect minority class instances. Models show poor diagnostic capability because they fail to identify patients accurately who carry a higher risk of disease. Scalability represents a key critical factor in these systems [8]. Modern healthcare data systems must analyze extensive datasets which emerge from electronic health records (EHRs) as well as wearable devices and medical imaging systems. Machine learning methods that follow traditional approaches experience difficulties in processing big data volumes effectively which calls for new analytical solutions for classifying and selecting relevant features. Data privacy and security present additional concerns. Healthcare data remains highly sensitive which requires predictive modeling applications to meet all relevant regulatory frameworks including HIPAA and GDPR. Federally imposed data regulations make complete datasets difficult to access thereby restricting joint research activities between institutions. The lack of transparent predictive models limits clinical deployment because healthcare professionals need clear explanations from decision-support systems.

Advanced machine learning techniques alongside big data analytics work to resolve existing difficulties. Dimension reduction through feature selection has become a necessary step for both dataset diminution and enhanced model performance [9]. Current approaches in feature selection combine statistical analysis with machine learning algorithms to discover optimal attributes that deliver appropriate accuracy alongside effective computational processing capabilities. Researchers used Synthetic Minority Oversampling Technique (SMOTE) along with its revamped versions to overcome the class imbalance challenge. SMOTE technologies create fake minority class data elements to build balanced training sets. The combination of resampling approaches alongside cost-sensitive learning techniques demonstrates promising results. Parallel distributed computing frameworks became necessary because of the scalability issues that needed resolution. The merger of machine learning algorithms operating within Apache Hadoop and Spark enables massive-scale data processing which drives exceptional computational efficiency. Real-time analysis becomes possible through these frameworks because they support processing healthcare data that enters the system continuously [10]. A breakthrough solution called federated learning stands as a game-changer for security and privacy enhancement. Model training occurs between multiple institutions through federation while protecting the raw patient data by maintaining institutional control. The combination of techniques known as differential privacy guarantees patient data confidentiality by preventing the disclosure of individual information when deriving insights from the data. Model interpretability motivates developers to establish explainable AI (XAI) methods that generate transparency into predictive model decision processes. The healthcare field has accepted these methods which enable clinicians to comprehend machine learning system predictions and build their trust in them. Models designed with explainable features in them achieve greater adoption rates in real-world clinical healthcare settings.

### Approaches in feature selection and data representation

A novel graph-based multiple kernel learning (GMKL) approach emerged through Hassanzadeh et al's work [11] to accomplish improved kernel-based algorithm performance. A low-rank graph representation enabled the approach to efficiently capture data structures on multiple scales which produced optimal discriminative features for classification. The method discarded excessive kernel searches by implementing projective directions that relied on graph theory structures hence providing improved computational performance. Maximizing data separation together with complex relationship processing emerged from their work on hyperspectral datasets of both Indian Pines and Pavia University when they conducted their experiments. Senan et al. [12] applied Recursive Feature Elimination (RFE) to find robust distinctive features within datasets through their research. Early diagnosis of chronic kidney disease through support vector machines (SVM), k-nearest neighbors (KNN), decision trees, and random forests was the main focus of this study. This strategy delivered precise prediction performance and simpler computing requirements. The authors from Azman et al. [13] integrated SVM-RFE onto multi-omics datasets to fight against the dimensionality curse. Changes in the selected subset quality were evaluated through SDAE and VAE deep learning classifiers which led to superior outcomes in lung cancer dataset classification. Random Forest Feature Selection (RFS) served as the method Channabasavaraju et al. [14] used to identify key features in diabetic heart disease datasets. Redundant and inconsistent features underwent removal to enhance the prediction accuracy for cardiovascular disease within diabetics according to their method. The approach established the possibility of connecting diabetes data for predictive modeling efforts with markers for heart disease as well as other health factors.

### Machine learning for disease classification

Huaping et al. [15] developed a deep neural network system for diagnosing diabetes along with type classification capabilities. The model successfully predicted diabetes diagnosis while providing distinction between Type 1 and Type 2 diabetes therefore guiding specific treatment strategies. High model accuracy resulted from using dropout regularization and binary cross-entropy loss functions as methods to overcome overfitting. García et al. [16] developed a data processing pipeline that performed information augmentation through VAEs combined with SAEs for feature augmentation. A classification model using convolutional neural networks (CNNs) showed great efficiency in diabetes prediction through its application on the Pima Indians Diabetes Database. Meryem et al. [17] developed a new handwriting evaluation approach for Parkinson's disease detection through analysis of Arabic handwriting executed directly on digital surfaces. The study proposed a feature selection method based on Recursive Feature Elimination with Cross-Validation (RFECV), evaluated using three estimators: Three machine learning prediction algorithms: Support Vector Machines, Decision Trees and Random Forest and their role in diabetes prediction using Pima Indians Diabetes Database. The selected features were input into the same models for detecting which method would best forecast PD instances. The investigation demonstrated how handwriting data aids in selecting significant features which support accurate classification.

Alalayah et al. [18] studied early diagnosis methods for Parkinson's disease (PD) by examining voice disorders. The study used machine learning methodologies including t-SNE and PCA for dimensionality reduction alongside RFE for feature selection. The processed dataset containing balanced records achieved accurate predictions of PD cases after performing hyperparameter optimization on various algorithms. The methodology demonstrated how effective disease classification depends on the combination of feature engineering with dimensionality reduction practices. According to Sabitha et al. [19] data preprocessing combined with feature selection and data augmentation produces better results for classification models. The Pima Indians Diabetes Database was utilized to demonstrate the effects these methods had on diabetes prediction performance. The study produced an organized assessment of machine learning models by testing versions with and without preprocessing methods.

### Hybrid and custom models

A customized hybrid model for medical dataset pattern recognition emerged from Rajagopal et al. [20]. After applying their novel normalization technique to preprocessing steps the model used decision-making algorithms to identify important variables. Special regularization techniques developed specifically for diabetes prediction improved the model's accuracy performance. By uniting RFE with artificial neural networks (ANNs) Balakrishnan et al. [21] developed an effective predictive model for diagnosing Alzheimer's disease. The combined research method achieved improved prediction accuracy alongside reduced computational demands which proves applicable to future diagnostic work in diabetes and other conditions. A deep autoencoder which combined multiple kernel learning (DAEMKL) served Zhou et al. [22] to predict microRNA-disease relationships. The model used miRNA and disease similarity networks alongside their integrated feature representations in a deep autoencoder to deliver an innovative solution for complex biomedical datasets. Naz et al. [23] applied machine learning methodologies to electronic health records to discover concealed patterns which aid in predicting diabetes onset in advance so big data analytics reveals its potential across healthcare.

Table 1 provides a comprehensive comparison of various methodologies, features, and challenges from recent studies addressing disease prediction and classification across diverse datasets and techniques. Current techniques demonstrate major gaps that need attention. Several machine learning and deep learning techniques have existed for disease prediction yet imbalanced datasets alongside high-dimensional data and inefficient feature selection remain as chief challenges. A majority of research focuses on accuracy-driven results without undertaking thorough analysis of computational system scaling capacity when processing extensive datasets. Domain-specific discoveries are frequently omitted from feature selection frameworks which results in less-than-optimal model performance. Present improvements for both accuracy and processing velocity remain absent from the existing methods which creates opportunities for dual optimization of these metrics. Our proposed Associative Kruskal Wallis Feature Selection model serves to address these shortcomings through its mechanism which selects balanced significant features thereby minimizing dataset imbalances and irrelevant attributes. Through combined local and global statistical insights, the model optimizes its feature selection precision. Strategic utilization of the MapReduce Poly Kernel Vector Classifier enables our method to handle big data sets efficiently while maintaining computational performance. Our model's dual emphasis on enhanced precision along with shortened computational time delivers both optimized forecasting operations and practical scalability for early disease identification in clinical settings.

**Table 1:** Features and Challenges of Existing Methodologies.

Author (Citation)	Methodology	Features	Challenges
Hassanzadeh et al. [11]	Graph-based MKL (GMKL) method with block diagonal representation for optimal feature separability.	Utilizes local and global data structures with superpixels and attribute profiles to generate discriminative kernels.	High computational cost in constructing the low-rank graph.
Senan et al. [12]	Recursive Feature Elimination (RFE) applied with SVM, KNN, Decision Tree, and Random Forest.	Identified significant attributes for CKD diagnosis using statistical analysis and machine learning models.	Missing data handling and computational overhead during RFE process.
Azman et al. [13]	SVM-RFE with SDAE and VAE classifiers for multi-omics lung cancer dataset.	Integrated multi-omics data with dimensionality reduction and feature selection methods.	Computational complexity of integrating and processing multi-omics datasets.
Channabasavaraju et al. [14]	Random Forest Feature Selection (RFS) for significant attribute selection in diabetic heart disease prediction.	Addresses redundancy and inconsistency in diabetic datasets using WHO guidelines for risk prediction.	Difficulty in managing redundant features and ensuring consistent data preprocessing.
Huaping et al. [15]	Deep neural network with dropout regularization and binary cross-entropy loss.	Transforms diabetes prediction into a classification task; distinguishes between Type 1 and Type 2 diabetes.	Requires extensive parameter tuning to maintain accuracy.
García et al. [16]	Data and feature augmentation using VAEs and SAEs with CNNs for classification.	Augments Pima Indians Diabetes Database for enhanced feature representation and classification accuracy.	Requires balanced datasets for optimal augmentation.
Meryem et al. [17]	RFECV with three estimators (SVM, Decision Tree, Random Forest) for Arabic handwriting analysis.	Extracted relevant features from handwriting for Parkinson's disease detection; compared classifier performances.	Requires optimization of RFECV estimators and consistent handwriting dataset annotations.
Alalayah et al. [18]	ML-based voice analysis for early Parkinson's disease diagnosis.	Balanced datasets using SMOTE; applied PCA and t-SNE for dimensionality reduction.	Hyperparameter tuning for ML models and achieving optimal feature subset selection.
Sabitha et al. [19]	Emphasizes data preprocessing, feature selection, and augmentation for diabetes diagnosis using the PIMA dataset.	Comparison framework for analyzing preprocessing impacts on classification models.	Managing diverse preprocessing techniques and ensuring robust feature selection.
Rajagopal et al. [20]	Hybrid model for pattern detection and customized regularization for diabetes prediction.	Novel normalization technique for skewed data; prioritized feature importance for effective classification.	Asymmetrical regularization method requires fine-tuning for variable dataset characteristics.
Balakrishnan et al. [21]	Combined RFE and ANN for Alzheimer's prediction.	RFE selects important features to reduce complexity; ANN improves accuracy and processing speed.	Challenges in handling non-linear relationships between variables.
Zhou et al. [22]	Deep autoencoder with multiple kernel learning (DAEMKL) for miRNA similarity and disease feature representation.	Constructs miRNA and disease similarity networks; applies reconstruction error for novel prediction of miRNA-disease associations.	High computational demand for similarity network construction and integration.
Naz et al. [23]	Diverse ML algorithms applied to healthcare data for diabetes prediction.	Utilized EHRs, omics, images, and text data for comprehensive early diagnosis of diabetes.	Handling large and heterogeneous healthcare datasets effectively.

**Method details**

## Methodology

**Fig. 1:**
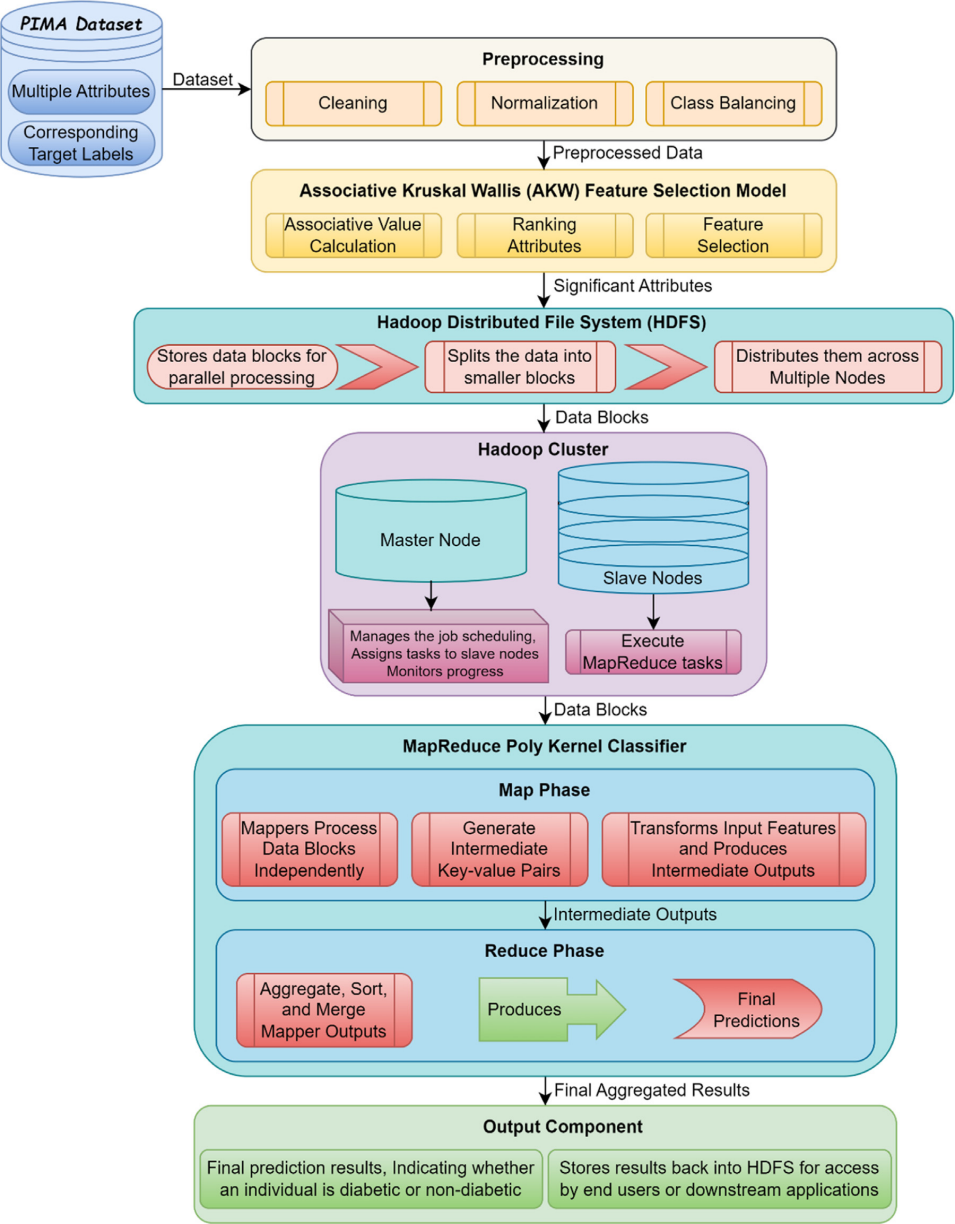
Block diagram of Associative Kruskal Wallis and MapReduce Poly Kernel (AKW-MRPK) Method.

Due to high sugar level in circulatory system, diabetes is said to occur and diabetes prediction at an early stage is said to be the need of an hour. Also, with the increase in the data size, significant feature selection or significant attribute selection plays a major role in early disease prediction. Therefore, our proposed work, MapReduce is used to process vast amounts of data (i.e., Big Data). By using Hadoop cluster which is capable of running MapReduce programs, first significant attributes are selected. Class imbalance also has an intense outcome on big data such that the conventional methods of examining these comprehensive datasets cannot give rise to the anticipated precise results. In our proposed work, Associative Kruskal Wallis is applied to the input dataset that addresses class imbalanced, contributing to significant features selection and therefore reducing the computational time involved. The block diagram of the Associative Kruskal Wallis and MapReduce Poly Kernel (AKW-MRPK) approach is shown in Fig. 1. As illustrated in the below-mentioned figure, with the PIMA Diabetes Dataset given as input, the proposed AKW-MRPK method diagnostically predict whether a patient has diabetes based on two models. The first model called Associative Kruskal Wallis Feature Selection chooses the significant attributes or features by addressing the class imbalance problem, therefore the computational time involved in feature selection is reduced. Next, a MapReduce Poly Kernel Vector Classifier model is applied to the significant attributes to speed up the evaluation process for large dataset size, therefore contributing to accuracy also. The proposed Associative Kruskal Wallis and MapReduce Poly Kernel (AKW-MRPK) method are discussed in the following sections.

### Associative Kruskal Wallis feature selection model

**Fig. 2:**
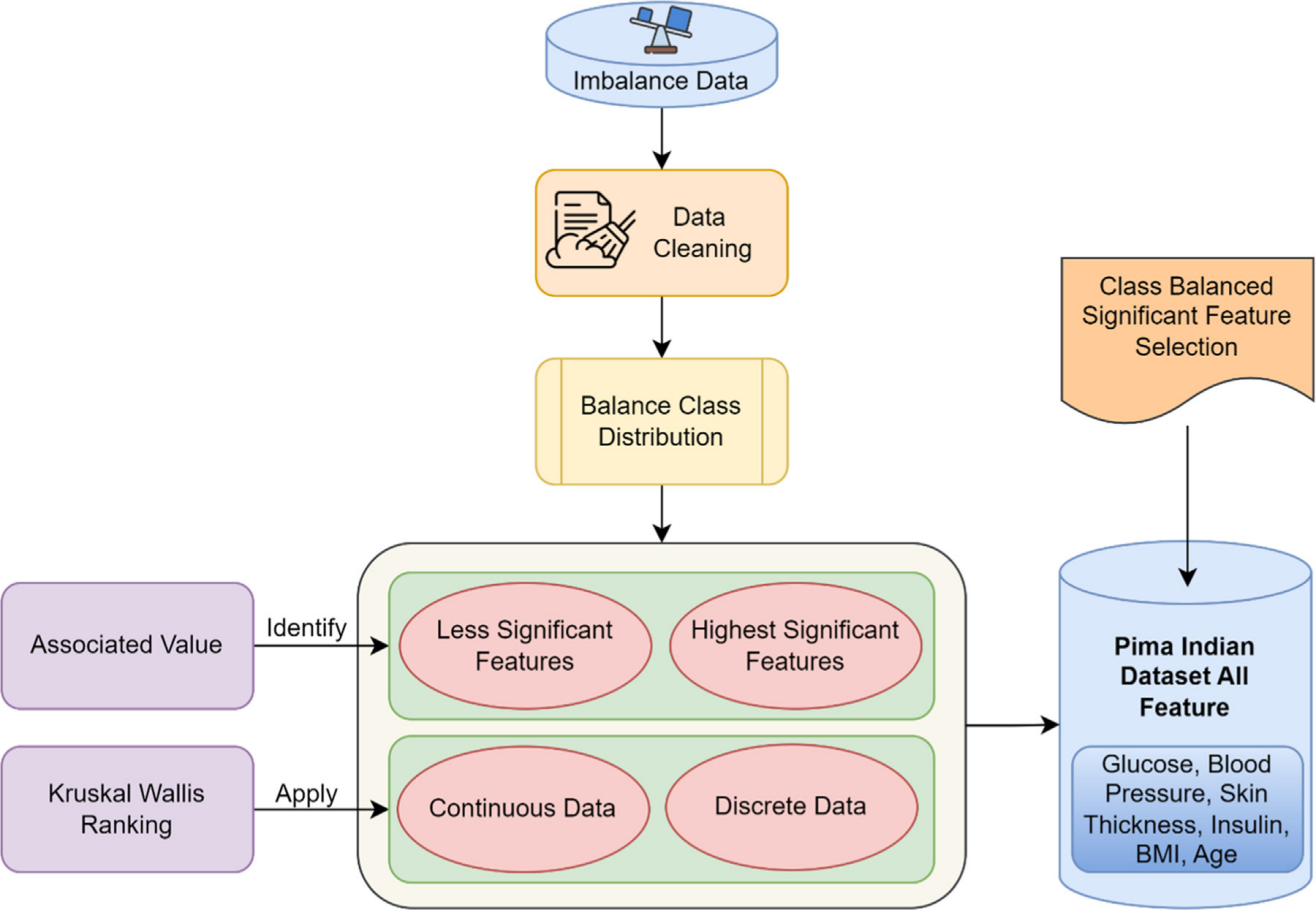
Block diagram of Associative Kruskal Wallis Feature Selection Model.

In this section, the choice of the optimal attributes or features from the large database based on the dataset sensitivity and the respective problem statement is included. The selection of optimal attributes for early diabetes prediction necessitates a comprehensive scanning of the attributes and rejecting the insignificant attributes. This is performed in this work by applying a value along with the Kruskal Wallis Ranking, therefore resulting in class balanced selection of significant feature. The block diagram of Associative Kruskal Wallis Feature Selection model is shown in Fig. 2. Classification of imbalanced data is said to be one of the most significant challenges biasing the vulnerabilities to the majority class target. Besides, the likelihood of the acquired target classes may be below the real numbers of the minority class in the dataset. To attain genuine classification along with sensitivities and data features, a model is proposed and also illustrated in the above-mentioned figure. With PIMA dataset provided as input, initially, the associative value is obtained as given below:

(1) $$AV=\;{\left[a_{N}-\sum_{i=1}^{N}a_{i}\right]}^{2}$$

As given in the above Eq. (1), the process of identifying the associative value is continued with all the attributes ‘$a_{i}$’ or features. Here, the values are compared with each attribute. If the difference is more than that of the other attribute, then the attribute is said to be less significant and the best attributes are associated with others. Next, variance, which is a measure of the spread of data items (i.e., consisting of both dependent and independent variables) of number ‘N’ from the mean is measured and given as below:

(2) $$\sigma^{2}=\frac{\sum {\left(a-\mu \right)}^{2}}{N}$$

Hence, for an input dataset with ‘$N=\{n_{1},\;n_{2},\;\ldots ..,n_{a}\}$’, the variance consists of both the discrete and continuous data items. If the variance of the continuous feature or attribute ‘a’is given by ‘$V_{a}$’ which is the expected value ‘EV’ of the square of the dispersion, for all the features of attribute ‘a’ with a mean of ‘μ’, the variance is given below:

(3) $$V_{a}=EV\left\{{\left(a-\mu \right)}^{2}\right\}$$

When the feature or attribute ‘a’ is continuous ‘C’ the variance is given below.

(4) $$V_{a}\left(C\right)=\sum_{i=1}^{n}\left({\left(a_{i}-\mu \right)}^{2}f\left(a\right)\right)$$

Besides, when the feature or attribute ‘a’ is discrete ‘D’ the variance is given below.

(5) $$V_{a}\left(D\right)=\;\int_{-\infty }^{+\infty }{\left(a-\mu \right)}^{2}\;f\left(a\right)\;da$$

Accordingly, when the entire dataset is considered, i.e., including both the continuous and discrete features, the population variance subject to probability density functions ‘f(a)’ is given below:

(6) $$V_{a}\left(T\right)=V_{a}\left(C\right)+V_{a}\left(D\right)+AV$$

Finally, the test statistics using Kruskal Wallis for both the discrete and continuous features is given below,

(7) $$H=V_{a}\left(T\right)\left[\frac{O_{i}\left(C\right)+O_{i}\left(D\right){\left[\left(AR_{i}-OR\right)\right]}^{2}}{{\left(OR_{ij}-OR\right)}^{2}}\right],\;where\;i,j=1,2,\ldots ,n$$

From the above Eq. (7), with the aid of ‘H’, considering the observation of continuous features ‘$O_{i}\left(C\right)$’, discrete features ‘$O_{i}\left(D\right)$’ and the average rank of all observations ‘$AR_{i}$’, the overall rank ‘OR’ comparison between independent samples of different or equal sample sizes are made and the significant features are selected accordingly. Algorithm 1

**Algorithm 1:** Associative Kruskal Ranking Feature Selection.

**Input**: Attributes ‘$A=\;a_{1},\;a_{2},\ldots ,a_{n}$’in Big Data dataset ‘D’
**Output**: Class balanced significant attribute
1: **Begin** 2: **For** each ‘D’and Attributes ‘A’ in Big Data dataset 3: Measure associative value using $AV=\;{\left[a_{N}-\sum_{i=1}^{N}a_{i}\right]}^{2}$ 4: Measure variance using $\sigma^{2}=\frac{\sum {\left(a-\mu \right)}^{2}}{N},\;while\;considering\;the\;whole\;data\;sources$ 5: Measure continuous variance using $V_{a}\left(C\right)=\sum_{i=1}^{n}{\left(a_{i}-\mu \right)}^{2}f\left(a\right)$ 6: Measure discrete variance using $V_{a}\left(D\right)=\;\int_{-\infty }^{+\infty }{\left(a-\mu \right)}^{2}\;f\left(a\right)\;da$ 7: Obtain class balanced significant attribute using $H=V_{a}\left(T\right)\left[\frac{O_{i}\left(C\right)+O_{i}\left(D\right){\left[\left(AR_{i}-OR\right)\right]}^{2}}{{\left(OR_{ij}-OR\right)}^{2}}\right],\;where\;i,j=1,2,\ldots ,n$ 8: **Return** (class balanced significant attribute) 9: **Endfor** 10: **End**

The Associative Kruskal Wallis Feature Selection model extends capability by incorporating its inherent strength for dealing with imbalanced class label distributions so it represents minority classes well through selection. The system accomplishes balanced representation through a merger of AV (Associative Value) evaluation with Kruskal Wallis statistical ranking to determine each feature's quantitative contribution to the class of interest. The model identifies essential attributes through systematic variance evaluation which includes both continuous and discrete characteristics and eliminates redundant or irrelevant features [24]. The identification of significant attributes occurs simultaneously with major data processing efficiency gains when working with large datasets such as the PIMA Indian Diabetes Database. Class-balanced feature selection simultaneously boosts downstream predictive accuracy and maintains a balanced approach to classification which prevents discrimination against the majority group. The methodology achieves additional strength by working with a parallel computing platform. The selection process divides into smaller manageable subsections through Hadoop MapReduce processing framework to enable simultaneous execution across multiple nodes of distributed systems. Large datasets benefit from significant speed-up through parallel computational processing which ensures their scalability alongside growing datasets. The system works by distributing data between different nodes which independently execute computations of associative values and variances and apply Kruskal Wallis ranking methods at the local level. This approach combines local result aggregation into global significance scores which enables real-time and large-scale healthcare applications to run successfully. By combining advanced statistical methods with parallelized big data processing, this research addresses two critical challenges in healthcare analytics: computational efficiency and the need for precise, unbiased predictions.

### MapReduce poly Kernel vector classifier model

For the prediction of diabetic disease, MapReduce model has been developed. Hadoop framework has been widely used among the implementations of computing model due to its open-source nature. Seyed Nima Khezr and Nima Jafari Navimipour [20] proposed a Hadoop cluster to a group of nodes associated together via Local Area Network (LAN), specifically utilized for Big Data storing and processing. Hadoop clusters communicate with a high-end machine, also referred to as the master. A distributing computing model is implemented by the master and slaves over distributed data storage for data management. The designing of the Hadoop Distributed File System (HDFS) is derived from the Google File System (GFS), where the architecture of HDFS architecture works according to master/slave architecture. Here, the Hadoop cluster comprises of a single name node and one or more data nodes. Besides, the single name node refers to metadata and data nodes refer to the actual data. In this work, MapReduce is utilized to exercise enormous data volume (i.e., big data) in a parallel fashion on large clusters. The MapReduce standard is one among of the efficient programming models for data-driven computing applications. Two key functions namely, Map function and Reduce function has a significant response on the MapReduce programming pattern. The Map function is used to create a set of intermediate key/value pairs by processing key/value pairs. On the other hand, the Reduce function combines all the indistinguishable values. Each Map and Reduce phase possess a key-value pair as input and output, with both the input and output stored in a Hadoop Distributed File System (HDFS).

**Fig. 3:**
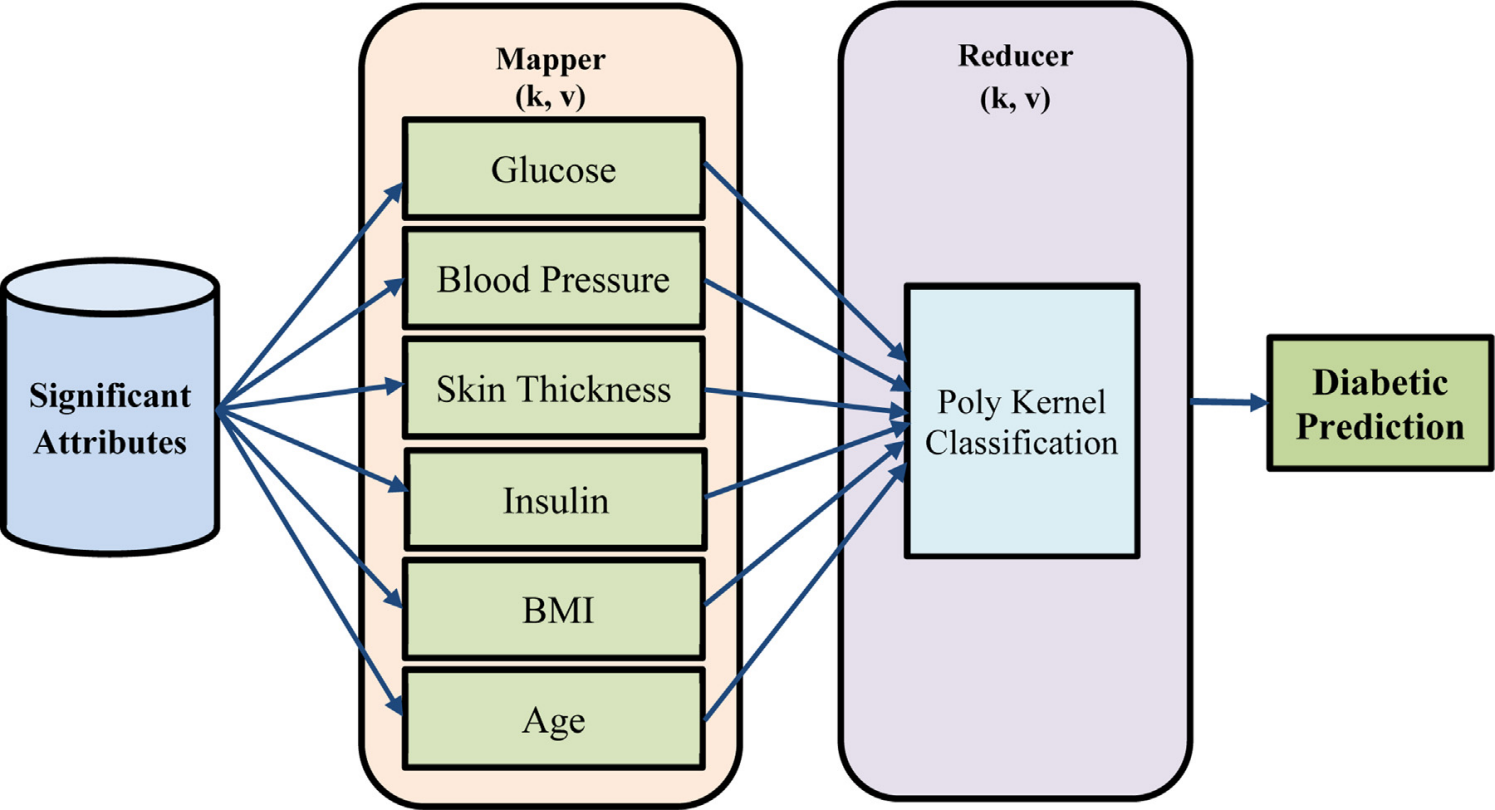
Block diagram of MapReduce Poly Kernel Vector Classifier model.

The input data (i.e., the significant attributes) is divided into small blocks of equal size and stored in the HDFS when a job (i.e., patient data) is submitted to a Hadoop cluster. Reducers in HDFS are claimed to sort, merge, and provide the final output results. The MapReduce workflow [20] for early diabetic prediction with PIMA dataset considered as input using the MapReduce Poly Kernel Vector Classifier model is shown in Fig. 3. The Hadoop framework as given in the above-mentioned figure is the prominent execution of the MapReduce model, permitting any applications to execute on large clusters. In this work, the healthcare system for diabetic prediction is developed. A single master node executes a Job tracker instance in the Hadoop MapReduce framework. The Job tracker instance acquires job requests from a client running a Task tracker instance on slave nodes. The Job tracker task also remains in furnishing the status in addition to the diagnostic information about the client. The tasks mentioned here are proposed to execute in JAVA so that several task instances are implicit to be performed in a parallel fashion. The proposed MapReduce Poly Kernel Vector Classifier model targets a situation in which support vector must predict a huge volume of testing data (i.e., either diabetic or non-diabetic). Let us consider a testing instance ‘$T_{i}=\left\{sa_{1},sa_{2},\;\ldots ,sa_{in}\right\}$’, where ‘$T_{i}$’ denotes an instance and ‘$a_{i}\in D$’ with ‘in’ representing the length of ‘$sa_{i}$’, also denoting the significant features or significant attributes. The input to be fed is capsulated in the form of ‘$\langle Instance_{i},\;Target_{i},type\rangle$’, with ‘$Instance_{i}$’ representing the input or support vectors, ‘$Target_{i}$’ forming the desirable output and ‘type’ denoting the training or test instance. To start with, the files or the class balanced significant features that comprise of the occurrences are stored into HDFS. As a result, each file, or class balanced significant features, contains all of the training instances as well as a portion of the testing instances, and the file number `N' represents the number of mappers to be used, while the file content (i.e. significant feature) remains the input to the MapReduce Poly Kernel Vector Classifier model.

Each mapper creates a support vector once the algorithm starts. As a result, the Hadoop cluster will have `N' support vectors. Each mapper parses to the next step by reading data in the form of 〈Instancei,Targeti,type〉. If the `type' field value is `train,' then the support vector machine is fed Instancei. The support vector machine uses non-linear mapping to translate the original data (i.e., significant qualities or features) into a feature space `FS' with high dimensionality, which is relevant to the kernel function selection. Given an input ‘$\{p_{i},q_{i}\},\;where\;i=1,2,\ldots ,N$’ where ‘$p_{i}\in \;sa_{1},\;sa_{2},\;\ldots ,sa_{N}$’ and ‘$q_{i}\in \left\{+1,-1\right\}$’, the classification decision is made as given below.

(8) $$q\left(p\right)=SIGN\left[\sum_{i=1}^{N}q_{i}\alpha_{i}*X\left(q,q_{i}\right)+b\right]$$

(9) $$Maximize\;\sum_{i=1}^{N}\alpha_{i}-\frac{1}{2}\sum_{i=1}^{N}\sum_{j=1}^{N}\alpha_{i}\alpha_{j}q_{i}q_{j}.X\;\left(p_{i},p_{j}\right)$$

$Subject\;to\;0\;\leq \alpha_{i}\leq RP$, After then, each mapper's support vector begins the hyperplane construction procedure using (10) that separates the data into two classes with the aid of the Regularization Parameter ‘RP’. The value of ‘RP’ is selected so that a trade-off between the margin and misclassification error is controlled.

(10) $$PK={\left(a*b+r\right)}^{d}$$

From the above Eq. (10), the polynomial kernel ‘PK’ is utilized that possess better response for high dimensional dataset along maintaining a reasonable trade-off. This is obtained using two different observations ‘a’ and ‘b’ about two different patients and selecting a coefficient of polynomial ‘r’ and degree of polynomial ‘d’ via cross-validation process. This polynomial coefficient and polynomial degree are chosen in such a manner to maintain high dimensional relationships. The support vector inputs, instance ‘i+1′. Repeat the above-mentioned two processes, i.e., mapping and hyperplane construction until all ‘$train^{\prime }$ instances are processed. By starting the mapping process, each mapper begins classifying instances titled as `test'. The efficiency is enhanced because each mapper only classifies a fraction of the full testing dataset. Finally, each mapper produces intermediate output in the form of ‘$\langle Instance_{i},\;O_{i}\rangle$’, where ‘$Instance_{i}$’ is the key and ‘$O_{i}$’is the ‘ith’ mapper's output. One reducer begins gathering and integrating all of the mappers' outputs. Finally, the reducer writes ‘$\langle Instance_{i},\;O_{i}\rangle$’ to HDFS. ‘$Instance_{i}$’denotes the final categorization findings in this case (i.e., either diabetes or non-diabetes) of ‘$O_{i}$’. Algorithm 2

**Algorithm 2:** MapReduce Poly Kernel Vector Classification.

**Input**: Significant Attributes ‘$sa_{1},\;sa_{2},\ldots ,sa_{in}$’, support vectors ‘N’
**Output**: Robust and early prediction
1: **Initialize** Significant Attributes ‘$sa_{1},\;sa_{2},\ldots ,sa_{in}$’, Regularization Parameter ‘RP’ 2: **Initialize** coefficient of polynomial ‘r’ and degree of polynomial ‘d’ 3: **Begin** 4: **For** each Significant Attributes ‘$sa_{1},\;sa_{2},\ldots ,sa_{in}$’ **MAP PHASE:** **5: Mapper - Data Mapping:** • **Input:** Significant attributes and support vectors. • **Action:** 1. For each training instance, map original data into the feature space using the SVM model. 2. Apply the kernel function (polynomial kernel in this case) to map data to a higher-dimensional space. 3. Compute support vectors, the decision function, and classification output. **6. Kernel Computation (within the Mapper):** • **Action:** 1. By Eqn. (11) compute polynomial kernel. **7. Mapper Output:** • Each mapper outputs an intermediate result: $\langle Instance_{i},\;O_{i}\rangle$ where $O_{i}$ is the predicted output (diabetic or non-diabetic) for instance i. **8. SHUFFLE AND SORT PHASE:** • The Hadoop framework sorts the intermediate key-value pairs from the map phase by the instance i (key). **REDUCE PHASE:** **9. Reducer - Result Aggregation:** • **Input:** Sorted key-value pairs from the map phase • **Action:** 1. The reducer collects all outputs from the mappers. 2. It aggregates the results (i.e., combines classification predictions). 3. Resolves conflicts and writes the final predictions back to HDFS. **10. Final Output:** • The reducer outputs the final prediction for each instance as: $\langle Instance_{i},\;O_{i}\rangle$. **11: End for** **12: End**

As given in the above-mentioned MapReduce Poly Support Vector Classification algorithm, for each significant attributes as input, the objective here remains in speeding up the performance with higher precision and accuracy. In order to achieve this, data mapping is performed based on the trade-off between the margin and misclassification error and utilizing polynomial kernel which in turn contributes to speed up the performance.

**Method validation**

## Results and discussion

Both Associative Kruskal Ranking Feature Selection and MapReduce Poly Support Vector Classification required execution on a Hadoop cluster with master-slave supervision. The implementation uses Hadoop version 1.1.2 which contains a master node alongside all other nodes functioning as slaves. Each node in the cluster including the master contains a Core2Duo CPU@2.20 GHz and 2GB of RAM with 100M/s network speed. The system runs Linux Ubuntu 16.04 LTS and links the nodes in a network named local area network (LAN). This implementation selected the SUN JAVA JDK 1.6.0_24 for processing MapReduce jobs because it provides efficient job execution alongside compatibility with Hadoop 1.1.2. Our system relies on three fundamental dependencies starting with Hadoop Common for its filesystem actions and including Hadoop HDFS for cluster data hosting and Hadoop MapReduce for parallel data execution. Three key third-party libraries became essential for this implementation: Apache Mahout to handle machine learning operations, Apache Commons Math for statistical functions and polynomial kernel applications and customized Java libraries for Kruskal Ranking and support vector classification processing. The configuration allows efficient dataset processing of large collections such as the PIMA Indian Diabetes Dataset while running within distributed computing setups.

Systematic optimization of MapReduce Poly Support Vector Classification implemented a systematic search for tuning of parameters, such as polynomial kernel degree (d) and regularization parameter (RP) values to achieve best algorithm performance. A grid search procedure optimized the regularization parameter (RP)that enables users to achieve appropriate margins while minimizing classification errors. Test results from five different RP levels starting at $10^{-3}$, $10^{-2}$, $10^{-1}$, 1, 10 enabled identifications of the best-performing RP value. The most accurate classification metrics emerged when testing each value on PIMA Indian Diabetes validation data subsets. The model determined the boundary complexity in a high-dimensional feature space through parameter adjustments to polynomial kernel degree d, where d ranged from 2 to 5. Cross-validation procedures were used to check model performance during testing at different degrees [25]. Experimental findings guided a set value for the polynomial coefficient (r) which achieved an appropriate balance between performance speed and prediction precision. The final parameters for the polynomial kernel and the regularization mechanism were selected because they demonstrated effective generalization during off-sample data evaluation. When replicating or adapting the model users should employ the same grid search method they use for parameter optimization. Users can adjust the values for RP and d and r depending on their dataset characteristics and computational power. Strict employment of cross-validation serves both to strengthen model robustness and protect from overfitting conditions [26]. These guidelines provide people with the tools they need to effectively adjust parameters when working with comparable datasets and applications.

An extensive preprocessing technique addressed class imbalance while identifying important attributes. The PIMA Indian Diabetes Dataset was handled for disparate values between classes by using oversampling and undersampling techniques alongside class weighting methods. SMOTE-based synthetic sample generation applied oversampling through minority class representation (diabetic cases) by creating new samples between existing case pairs. For example, through SMOTE's methodology the system generated synthetic data pair [3.5, 125, 77.5, 26.5] from two diabetic samples [4, 120, 80, 25] and [3, 130, 75, 28]. Sample size reduction through undersampling allowed us to balance the diabetic (minority class) and non-diabetic samples (majority class). The loss function of this classifier contained class weighting to ensure both classes would maintain their equal weighting during computations [27]. The Associative Kruskal Ranking Feature Selection Algorithm served to determine essential attributes for selection. The feature selection process started by calculating the attributes' associative value through the equation $AV\;=\;{\left[a_{N}-\;\sum_{i=1}^{N}a_{i}\right]}^{2}$ while $a_{i}$ stands for attribute values. The assigned Attribute Value for this set of values at [100, 120, 130] becomes 6400. The assessment of attribute significance relied on variance metrics where both continuous and discrete data types were examined. Continuous variance calculations for [4,6,8] values equaling μ = 6 resulted in a value of $\sigma^{2}$ = 2.67. The Kruskal Ranking method evaluated attributes which demonstrated significant variance between values and persistent associations resulting in selection of plasma glucose and BMI as priority attributes. A final set of attributes underwent adjustment based on class balancing metrics to achieve equal class representations. Procedure-led data normalization transformed the dataset into an optimized training format for MapReduce Poly Kernel Vector Classifier implementation.

### Dataset description

The PIMA Indian Diabetes Dataset used in this study is publicly available on Kaggle and can be accessed at https://www.kaggle.com/datasets/uciml/pima-indians-diabetes-database. This database includes diverse features that support diabetes prediction analyses which will act as the fundamental building block for our proposed method. Academic researchers can obtain and preprocess this dataset following study guidelines to execute their own experiments. Additional investigations and validation can be performed through Hadoop by operationalizing the defined workflow that incorporates Associative Kruskal Wallis feature selection and MapReduce Poly Kernel classification procedures. The PIMA Indian Diabetes Dataset contains information on female patients in the PIMA Indian population who are at least twenty-one years old, as retrieved from the UCI machine learning repository, which was previously owned by the National Institute of Diabetes and Digestive and Kidney Diseases.

The dataset has a total of 768 instances with eight risk factors. They are divided into two groups: diabetes and non-diabetic. Table 2 lists the parameters: number of pregnancies, two-hour plasma glucose concentration in an oral glucose tolerance test, diastolic blood pressure, triceps skinfold thickness, two-hour serum insulin, body mass index, diabetes pedigree function, and age.

**Table 2:** PIMA Indian Diabetes Dataset.

Attribute number	Attribute
A1	Number of times Pregnant
A2	2- Hours Plasma glucose concentration
A3	Diastolic blood pressure (BP)
A4	Triceps skin fold thickness
A5	2- Hours Serum insulin
A6	Body mass index (BMI)
A7	Diabetes pedigree function
A8	Age (years)

### Evaluation measures

A comparison of the proposed method's performance with supervised machine learning algorithms and machine learning algorithms in Hadoop based clusters are conducted based on metrics, computational time, speedup, and accuracy for various numbers of patients.

#### Scenario 1: computational time

The amount of time it takes to predict diabetes is referred to as computational time. If the consumed computational time is decreased, then better and early diagnosis can be achieved and therefore lesser is the mortality rate also. The classification time is mathematically evaluated as given below:

(11) $$CT=\;\sum_{i=1}^{n}P_{i}*Time\;\left[q_{i}\right]$$

**Fig. 4:**
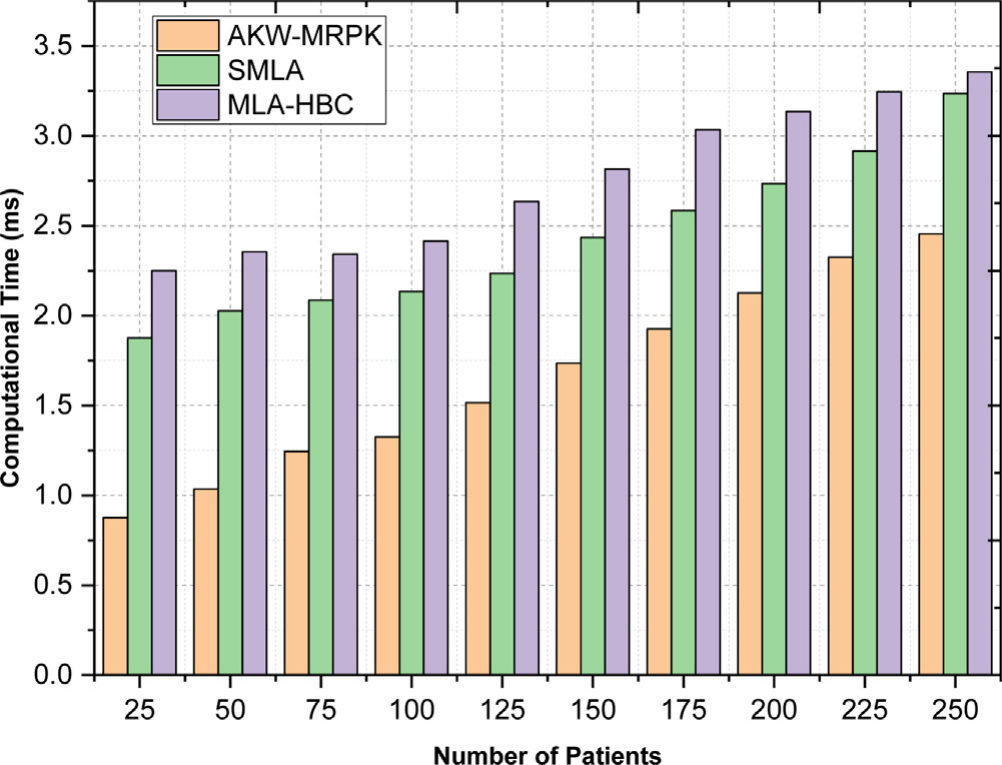
Computational Time Comparison of Three Diabetes Prediction Method.

From the above Eq. (11), the computational time ‘CT’ is measured based on the frequency or number of patient's ‘$P_{i}$’ diagnostic measurement and the time consumed in arriving at the result ‘$Time\;[q_{i}]$’ respectively. CT is measured in milliseconds (ms). Table 2 shows the comparison of computation time using the AKW-MRPK method to compare the results of computational time involved in the prediction of diabetic disease using supervised machine learning algorithms and machine learning algorithms in Hadoop based clusters. Fig. 4 shows the computational time with respect to 250 different numbers of patients. The 250 different patient data are obtained from PIMA Indian Diabetes Dataset. This dataset includes 21 years old female. It can be noticed when the data size was lesser (i.e., with a smaller number of patients), the computational time will be lesser and higher with the increase in data size. This is due to the reason that increasing the data size, increases the number of patients to be monitored and therefore, the computational time involved in diagnosing the disease also gets increased. However, the computational time using AKW-MRPK method was better compared to existing works. Table 3

**Table 3:** Comparison of Computational Time.

Number of patients	Computational time (ms)		
	AKW-MRPK	Supervised Machine Learning Algorithm	Machine Learning Algorithms in Hadoop-based Clusters
**25**	0.875	1.875	2.25
**50**	1.035	2.025	2.355
**75**	1.245	2.085	2.341
**100**	1.325	2.135	2.415
**125**	1.515	2.235	2.635
**150**	1.735	2.435	2.815
**175**	1.925	2.585	3.035
**200**	2.125	2.735	3.135
**225**	2.325	2.915	3.245
**250**	2.455	3.235	3.355

This is evident from the simulation with ‘25′ patients observed, the time consumed in obtaining the desired output as either diabetes or non-diabetes being ‘0.035ms', ‘0.075ms' using the work done by (give authors name) and ‘0.090ms’ using the work done by (give authors name). This is because of the application of Associative Kruskal Ranking Feature Selection algorithm. By applying this algorithm, two critical concerns have been addressed and those are obtaining the significant feature and addressing the class imbalance problem. With these two concerns, significant features were extracted by AKW-MRPK method and were found to be reduced by 33 % compared to supervised machine learning algorithms and 41 % compared to machine learning algorithms in Hadoop based clusters.

#### Scenario 2: speedup

The effectiveness and scalability of the proposed AKW-MRPK method in terms of speedup are defined in following formulas.

(12) $$Speedup=\;\frac{CT\;on\;one\;computer}{CT\;on\;cluster}$$

**Fig. 5:**
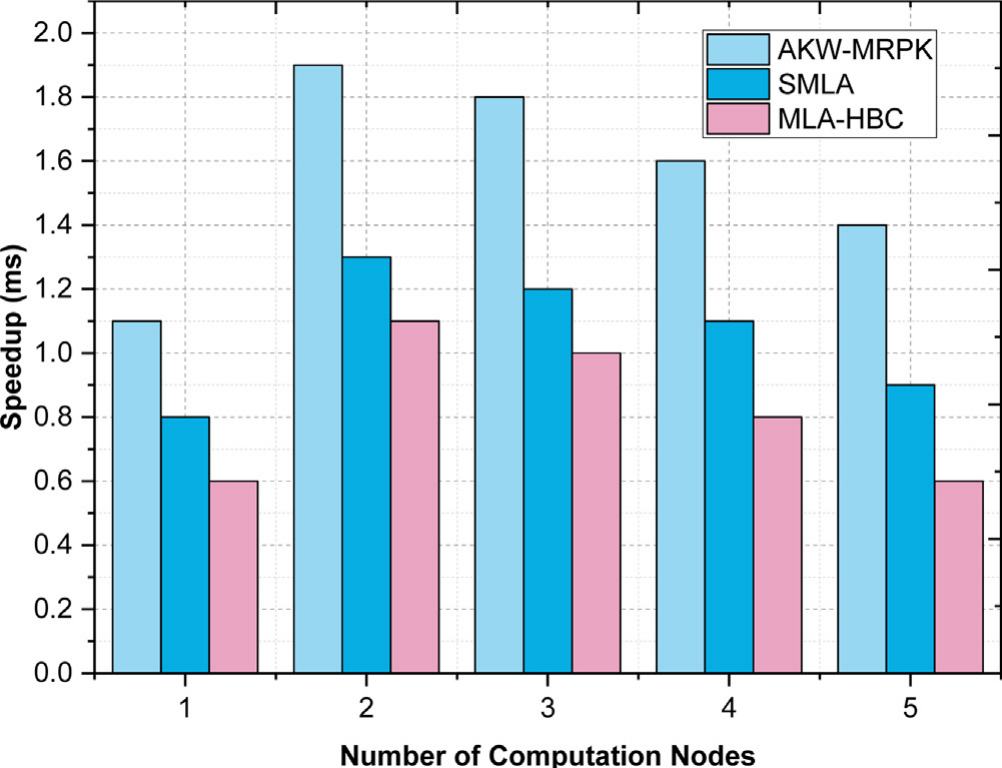
Speedup Comparison of three Diabetes Prediction.

From the above Eq. (12), the speedup is measured based on the computation time on one computer ‘CTononecomputer’ to the computation time on cluster ‘CToncluster’. Table 4 is the comparison table using the AKW-MRPK method to compare the results of speedup, supervised machine learning algorithms and machine learning algorithms in Hadoop based clusters. To evaluate the process of speedup, the number of training dataset (i.e. PIMA Indian Diabetes Dataset) as persistent and the computing nodes in the clusters were increased. Obviously, the parallel algorithm adopted using Hadoop Cluster and Polynomial Kernel in the proposed method demonstrates linear speedup. But, as far as linear speedup is concerned, due to the increase in the communication cost and parallel performance, an increase in speedup is a challenge to achieve. Hence, the speedup evaluation in the proposed method has been performed on the training dataset with varying sizes and systems. The number of computing nodes was kept to be varied between 1 and 5. The amount of training samples rises from 25 to 250. From the Fig. 5, it is illustrative that the proposed method's speedup performance is better than supervised machine learning algorithms and machine learning algorithms in Hadoop based clusters. This is because of the SVM model's application of Polynomial Kernel, which addresses exponential incoming nodes (with each node potential of predicting the diabetes disease) in a better manner. The reason behind this, the time to process lesser dataset with the significant feature is not prominent associated with the time spent by communication and task arrangement. However, as the training dataset grows larger, computing time becomes more important, improving the speedup. Hence, the proposed AKW-MRPK methods treat large datasets in an efficient manner with 56 % improvement over supervised machine learning algorithms and 78 % improvement over machine learning algorithms in Hadoop-based clusters.

**Table 4:** Comparison of Speedup Time.

Number of computation nodes	Speedup (ms)		
	AKW-MRPK	Supervised Machine Learning Algorithm	Machine Learning Algorithms In Hadoop-Based Clusters
**1**	1.1	0.8	0.6
**2**	1.9	1.3	1.1
**3**	1.8	1.2	1
**4**	1.6	1.1	0.8
**5**	1.4	0.9	0.6

#### Scenario 3: accuracy

Prediction accuracy refers to the measurement of how far the proposed method is efficient in early diagnosis. It is mathematically evaluated as given below.

(13) $$A=\frac{Corr_{p}}{O_{Samples}}*100$$

**Fig. 6:**
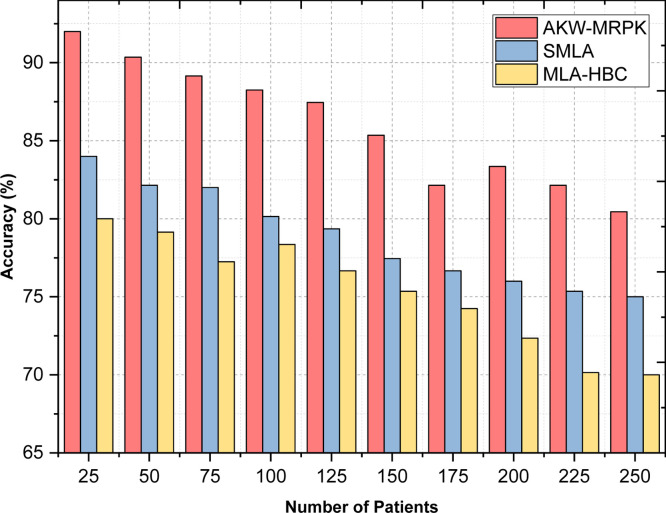
Accuracy Comparison of Three Diabetes Prediction.

In other words, prediction accuracy measures the percentage ratio of correct prediction ‘$Corr_{p}$’ to the overall samples ‘$O_{Samples}$’ considered for experimentation. It is measured in terms of percentage (%). Table 5 is the comparison table using the AKW-MRPK method to compare the results of accuracy, supervised machine learning algorithms and machine learning algorithms in Hadoop-based clusters. Fig. 6, given above illustrates the prediction accuracy with respect to 250 different numbers of patients obtained at different time intervals with the attributes as number of pregnancies, BMI, insulin level, age and so on. The accuracy reduces significantly to some extent as the number of patients increases, as shown in the above table. This is because when the number of patients increases, first features are selected from which significant features are obtained. Then, the actual prediction is made based on the Hadoop cluster Polynomial Kernel model. However, from the simulations, it's identified that considering 25 patients for predicting the diabetic disease, 23 patients were correctly predicted using AKW-MRPK when compared to 21 patients using supervised machine learning algorithms and 20 patients using machine learning algorithms in Hadoop-based clusters.

**Table 5:** Comparison of Accuracy.

Number of patients	Accuracy (%)		
	AKW-MRPK	Supervised Machine Learning Algorithm	Machine Learning Algorithms In Hadoop-Based Clusters
**25**	92	84	80
**50**	90.35	82.15	79.15
**75**	89.15	82	77.25
**100**	88.25	80.15	78.35
**125**	87.45	79.35	76.66
**150**	85.35	77.45	75.35
**175**	82.15	76.66	74.25
**200**	83.35	76	72.35
**225**	82.15	75.35	70.15
**250**	80.45	75	70

**Fig. 7:**
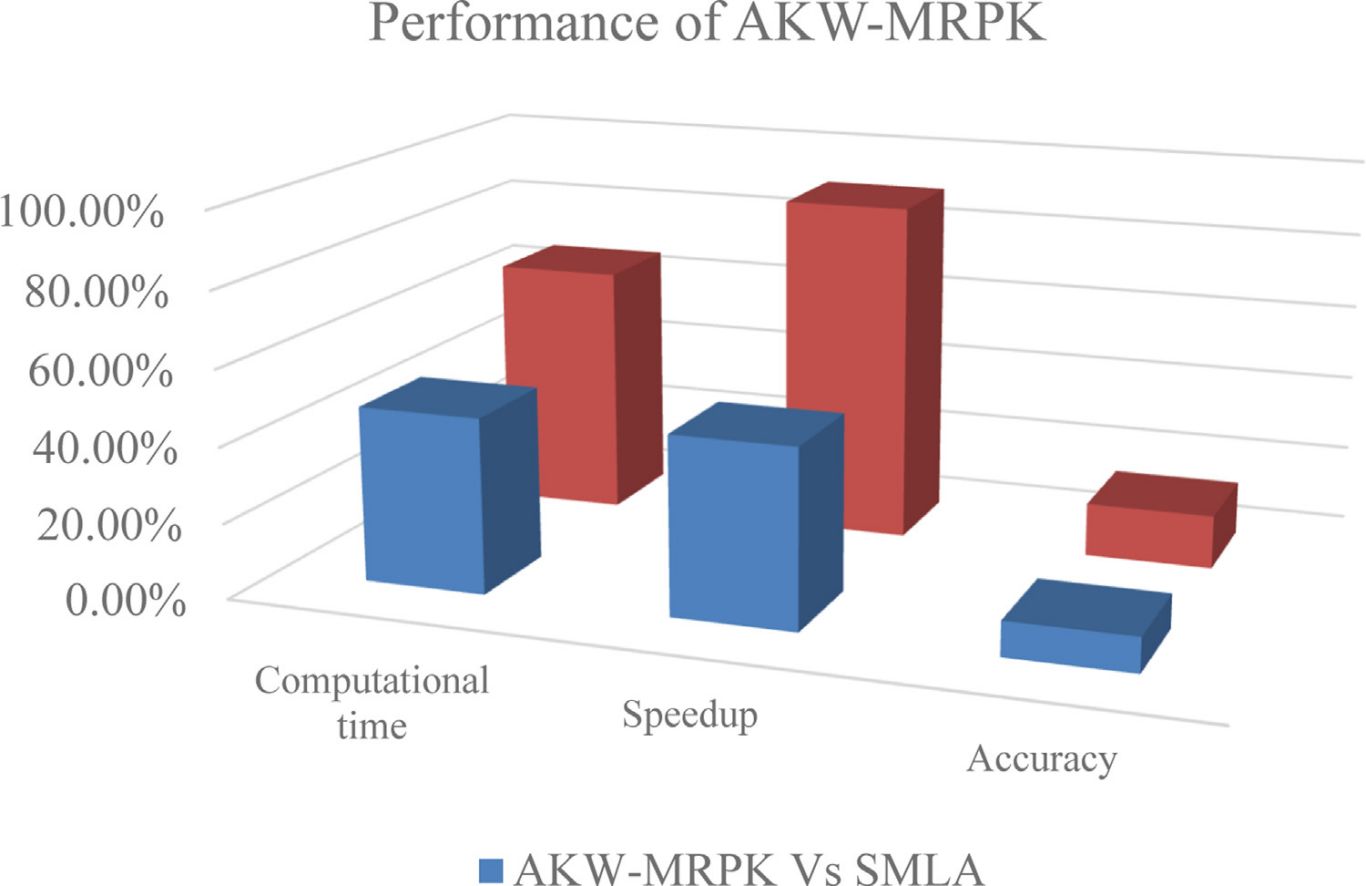
Performance of AKW-MRPK.

Least significant attributes are eliminated because the value difference is more than other attributes whereas highest significant attributes are considered. As the significant features obtained above consist of both dependent and independent variables, variance is measured. The significant features or attributes involves both the continuous and discrete data and by separating the data into continuous and discrete results in computationally efficient feature selection. Feature selection uses Kruskal Wallis ranking for both the discrete and continuous feature. With the above values arrived for discrete and continuous function, pregnancies and diabetes pedigree are ranked least and with this the other features or attributes are selected as the overall significant features. They are glucose, Blood Pressure, Skin Thickness, Insulin, BMI and Age. Early prediction is said to be done using MapReduce Poly Support Vector Classification. In conventional PK the value of ‘r’, is either ‘≥0or=0′. In this work, the value of ‘$r^{\prime }$ considered as coefficient of polynomial, because here, the coefficient values differ according to the number of significant features obtained. This is due to the application of first associative value along with the Kruskal Wallis that first eliminates the insignificant attributes. After this, the prediction is completed via Polynomial Kernel SVM classification, which improves the accuracy using AKW-MRPK to 9 % compared to supervised machine learning algorithms and 14 % compared to machine learning algorithms in Hadoop-based clusters. From the Fig. 4, Fig. 5, Fig. 6, it is clearly evident that the proposed AKW-MRPK outperforms the other algorithms. The percentage improvement in computation time, speedup and accuracy is presented in Fig. 7.

## Discussion

In this study, the proposed Associative Kruskal Wallis and MapReduce Poly Kernel (AKW-MRPK) method demonstrates its effectiveness in early disease prediction, specifically for diabetes. The approach integrates feature selection using the Associative Kruskal Wallis model and the parallelization of polynomial kernels using the MapReduce framework, ensuring both scalability and computational efficiency. By reducing the data size through feature selection, the AKW-MRPK method focuses on the most significant attributes, which improves both computational time and predictive accuracy. The experimental results on the PIMA Indian Diabetes Dataset show that the AKW-MRPK method outperforms traditional supervised machine learning algorithms and machine learning techniques in Hadoop-based clusters. Scenario 1 reveals a significant improvement in computational time, particularly with larger datasets. As the number of patients increases, the efficiency of AKW-MRPK in managing larger datasets becomes clear, offering a speedup that addresses the challenges of handling extensive datasets. Scenario 2 highlights the speedup achieved through parallel processing, where AKW-MRPK demonstrates better linear speedup as compared to other approaches. Scenario 3 emphasizes the accuracy of the method, showing a steady and consistent improvement in early prediction over traditional models. The use of Polynomial Kernel Support Vector Classification has been key in addressing the exponential growth in data, enabling AKW-MRPK to deliver precise and reliable predictions even with increased data complexity. The key strengths of the proposed method are its ability to handle large datasets efficiently, maintain accuracy with increased data, and reduce computational time. The parallelization aspect also adds scalability, making it suitable for real-world applications, especially in healthcare, where timely diagnosis is critical.

### Generalizability and broader applications

The framework design based on Associative Kruskal Ranking Feature Selection algorithm in combination with MapReduce Poly Kernel Vector Classifier functions as a multitudinous system for multiple datasets including beyond the PIMA Indian Diabetes dataset. The algorithm applies universal classification principles with fundamental feature determination protocols thus enabling flexible application throughout multiple sectors including healthcare domains. The methodology requires adaptation to new datasets and medical applications through basic modifications focused on preprocessing and feature selection steps to address each domain-specific data pattern and characteristic. When applying this framework to a cardiovascular dataset like heart disease prediction data the attributes blood pressure results ECG readings resting heart rate and cholesterol levels would serve as the PIMA dataset substitutes. Following preprocessing the Associative Kruskal Ranking algorithm detects which predictors most strongly influence cardiovascular risk thus maximizing model effectiveness. The technology can apply specifically to cancer prediction models which use oncology datasets for predicting recurrence probabilities. A framework could process tumor measurements and genetic aspects together with patient age data supplemented by histological results to generate predictions. The data preprocessing process includes methods to handle unedited data points while harmonizing feature scales together with specific solutions for balancing uneven outcomes typical of cancer samples. The refined features obtained through preprocessing enable the MapReduce Poly Kernel Vector Classifier system to classify large oncology datasets effectively. Future applications of this work should integrate domain-specific knowledge throughout preprocessing tasks alongside feature selection methods. The framework can achieve improved results with unstructured data such as medical images and genomic sequences when it blends advanced deep learning feature representation models with current infrastructure. Through customized preprocessing methods and kernel tuning according to individual dataset properties this framework generates dependable medical predictions across numerous healthcare domains.

## Limitations

The proposed method by combining Associative Kruskal Ranking Feature Selection and MapReduce Poly Kernel Vector Classifier achieved promising outcome results. However, certain challenges present opportunities for further optimization. Scalability with extremely large datasets remains a critical consideration, as the computational load on the Hadoop framework and the MapReduce algorithm can increase significantly with data size. Research directions focusing on distributed computing optimizations must be pursued which include Apache Spark's utilization for in-memory computation and GPU-accelerated processing for improved performance. The deployment of models faces implementation difficulties when computational resources within a system are restricted. The development of light-weighted algorithms is possible through model compression techniques such as pruning and quantization and knowledge distillation for lower computational demands without sacrificing model performance. The current feature selection strategy functions only with structured datasets when using the PIMA Indian Diabetes Dataset. This method must evolve to accept unstructured and semi-structured datasets by implementing adaptable computational systems that process multiple data forms. The process for manually adjusting parameters such as polynomial kernel degree and regularization parameter can become unproductive when dataset complexity grows. Bayesian optimization and grid search automation technologies provide optimal performance results across multiple datasets. The framework's future application in dynamic big data environments can be achieved through improvements which will boost the framework's robustness and scalability along with increased efficiency.

## Conclusion & future work

Diabetes has been considered as one of the significant heterogeneous types of disease and it affects larger volume males and females invariably. However, early-stage detection of disease helps to improve the mortality of life through better treatment. In this research work, an Associative Kruskal Wallis and MapReduce Poly Kernel (AKW-MRPK) method was presented to detect diabetes at an early stage. Initially, an Associative Kruskal Wallis Feature selection model is proposed to address the class imbalance issue and hence the significant features are selected. Subsequently, MapReduce Poly Kernel Vector Classifier framework is applied to the obtained significant attributes to speed up or accelerate the process as well as to increase the accuracy. Comparative analysis has accomplished well among the proposed prediction methods like supervised machine learning algorithms and machine learning algorithms in Hadoop-based clusters. According to the experiment results, the suggested methodology AKW-MRPK performs in terms of process speedup, computational time and precision. In future, the proposed feature selection model may further be extended for reducing complexity by removing redundancy, missing value and null values of features and it turn, it will reduce the processing complexity of big data. The performance accuracy will also increase through combining few technical indicators with the model. In future, the proposed work may also be used in other benchmark datasets with more instances and attributes for evaluating the performance of diabetic disease prediction. In addition, the optimization techniques may be used in future to extract the significant features from the large dataset for providing better results.

## Ethics statements

In this Manuscript no, human participants or animals their data or biological material, are not involved.

## CRediT author statement

**R. Ramani:** Data curation, Software, Validation, Field study. **S. Edwin Raja:** Visualization, Investigation, Software. **Dhinakaran D:** Conceptualization, Methodology, Writing-Original draft preparation. **S. Jagan:** Methodology, Writing-Reviewing and Editing. **G. Prabaharan:** Writing-Reviewing and Editing, Investigation.

## Declaration of competing interest

The authors declare that they have no known competing financial interests or personal relationships that could have appeared to influence the work reported in this paper.

## Data Availability

No data was used for the research described in the article.
